# Creation of an iPSC-Based Skeletal Muscle Model of McArdle Disease Harbouring the Mutation c.2392T>C (p.Trp798Arg) in the *PYGM* Gene

**DOI:** 10.3390/biomedicines11092434

**Published:** 2023-08-31

**Authors:** Victoria Cerrada, Inés García-Consuegra, Joaquín Arenas, M. Esther Gallardo

**Affiliations:** 1 Grupo de Investigación Traslacional con Células iPS, Instituto de Investigación Sanitaria Hospital 12 de Octubre (imas12), 28041 Madrid, Spain; 2 Laboratorio de Enfermedades Mitocondriales y Neuromusculares, Instituto de Investigación Sanitaria Hospital 12 de Octubre (imas12), 28041 Madrid, Spain; 3 Centro de Investigación Biomédica en Red de Enfermedades Raras (CIBERER), 28029 Madrid, Spain

**Keywords:** induced pluripotent stem cell, iPSCs, McArdle disease, *PYGM*, disease modelling, skeletal muscle differentiation

## Abstract

McArdle disease is a rare autosomal recessive condition caused by mutations in the *PYGM* gene. This gene encodes the skeletal muscle isoform of glycogen phosphorylase or myophosphorylase. Patients with McArdle disease have an inability to obtain energy from their muscle glycogen stores, which manifests as a marked exercise intolerance. Nowadays, there is no cure for this disorder and recommendations are intended to prevent and mitigate symptoms. There is great heterogeneity among the pathogenic variants found in the *PYGM* gene, and there is no obvious correlation between genotypes and phenotypes. Here, we present the generation of the first human iPSC-based skeletal muscle model harbouring the second most frequent mutation in *PYGM* in the Spanish population: NM_005609.4: c.2392T>C (p.Trp798Arg). To this end, iPSCs derived from a McArdle patient and a healthy control were both successfully differentiated into skeletal muscle cells using a small molecule-based protocol. The created McArdle skeletal muscle model was validated by confirming distinctive biochemical aspects of the disease such as the absence of myophosphorylase, the most typical biochemical feature of these patients. This model will be very valuable for use in future high-throughput pharmacological screenings.

## 1. Introduction

Glycogen storage diseases are inherited inborn errors of carbohydrate metabolism. These diseases are caused by the lack of an enzyme needed to store glucose into glycogen or break down glycogen properly [1]. This group of pathologies is classified numerically in the order of recognition and identification of the enzyme defect generating the disorder.

McArdle disease, or glycogen storage disease type V (OMIM 232600), is a rare autosomal recessive disorder caused by mutations in the *PYGM* gene. This gene encodes for the muscle isoform of glycogen phosphorylase or myophosphorylase. This enzyme initiates glycogen breakdown by removing α-1,4 glucosyl units from the glycogen outer branches producing the liberation of glucose-1-phosphate in muscle fibres. As a result, the carbohydrate metabolism of the skeletal muscle is affected, and energy cannot be generated from the muscle glycogen stores. Therefore, patients with McArdle disease suffer from exercise intolerance with muscle cramps, fatigue, myalgia, contractures and even rhabdomyolysis, as reflected by a massive release of muscle proteins into the blood such as creatine kinase or myoglobin [2]. To date, more than 150 different mutations in the *PYGM* gene have been reported including non-sense mutations, deletions and missense mutations and there is phenotypic heterogeneity among patients [3]. Indeed, according to epidemiologic data, there is no correlation between the genotype and phenotype in patients with this disorder [4].

Up to the present time, there is no effective treatment for McArdle disease. Some studies have shown that pharmacological or nutritional supplements can alleviate symptoms [5] and paradoxically, supervised light-moderate physical exercise seems to be the most promising therapy [6]. Furthermore, models on which research has been based to date are mainly animal models, either spontaneous or generated in the laboratory, and despite being valuable for expanding knowledge about the disease, they are not suitable for performing high-throughput drug screenings.

Induced pluripotent stem cell (iPSC)-based cellular models represent a powerful tool for the generation of patient-specific disease models. iPSCs are directly generated from adult cells. These cells are reprogrammed to a pluripotent state by the introduction of the Yamanaka factors [7]. The possibility to potentially differentiate these iPSCs into whatever cell type present in the body is a cornerstone of personalized medicine. Since they can be generated from any healthy person or patient, iPSCs are considered a valuable resource for regenerative medicine to replace damaged or diseased tissues. In addition, they have been demonstrated to be very useful for modelling human diseases in the affected target cell type, allowing for their use as a platform to perform high-throughput drug screenings.

Recently, our group reported the generation of the first human in vitro skeletal muscle model of McArdle disease based on iPSC technology [8]. This model was obtained from a patient harbouring the most common genetic defect in the *PYGM* gene described in the Caucasian population: NM_005609.4: c.148C>T (p.Arg50Ter). In this article, we present a human iPSC-based skeletal muscle model of McArdle disease carrying the second most frequent mutation in *PYGM* in the Spanish population: NM_005609.4: c.2392T>C (p.Trp798Arg). The results obtained show that patient-derived skeletal muscle cells mimic distinctive biochemical features of the disease such as the absence of myophosphorylase.

In summary, to the best of our knowledge, this is the first human model of McArdle disease carrying the missense mutation in the *PYGM* gene: NM_005609.4: c.2392T>C (p.Trp798Arg). It will be very useful to explore this model as a platform to perform high-throughput drug screenings to search for a personalized treatment of this disease.

## 2. Materials and Methods

### 2.1. Biological Samples

#### iPSC Lines

The iPSC control line NSV44.1 was previously established in our group by reprogramming healthy human dermal fibroblasts using non-integrative Sendai viruses for the delivery of the Yamanaka factors [9]. McA2.7 is an iPSC line created from the PBMCs of a McArdle patient harbouring the homozygous mutation in the *PYGM* gene: NM_005609.4: c.2392 T>C (p.Trp798Arg) [10].

### 2.2. iPSC Culture

iPSCs were cultivated on Matrigel hESC-qualified matrix (Corning, Corning, NY, USA, 354277) coated dishes with daily mTeSR1^TM^ medium changes (Stemcell Technologies, Vancouver, BC, Canada, 85850) in a cell incubator at 37 °C and 5% CO_2_. When 70% confluence was reached, the iPSCs were split into a 1:4 ratio using ReleSR^TM^, following the instructions of the manufacturer (Stemcell Technologies, Canada, 100-0484).

### 2.3. Skeletal Muscle Differentiation of iPSCs

To achieve skeletal muscle differentiation from the iPSCs, a combination of previously published protocols were followed. On the one hand, to differentiate iPSCs into myogenic progenitors (primary differentiation), we followed the protocol described by Chal et al. [11]. On the other hand, to differentiate myogenic cells into mature myofibres (final differentiation), we performed the modifications and guides proposed by Al Tanoury et al. [12]. Both methods are based on the addition of small molecules that activate and inhibit different pathways involved in the embryonic development of skeletal muscles.

#### 2.3.1. Primary Differentiation

To begin the differentiation process, cells must have reached about 70% confluence and iPSCs cultures should have consisted of healthy good-looking colonies, with bright borders and a compact aspect. Subsequently, the iPSCs were treated for 2 h with 10 μM of Rock inhibitor Y27632 (Miltenyi, Bergisch Gladbach, Germany, 130-103-922). After this time, cells are dissociated with Accutase (ThermoFisher, Waltham, MA, USA, A1110501) and 1.2 × 10^5^ cells per well were plated in a 12-well plate with the mTeSR1^TM^ medium supplemented with the Rock inhibitor.

**Figure 1:**
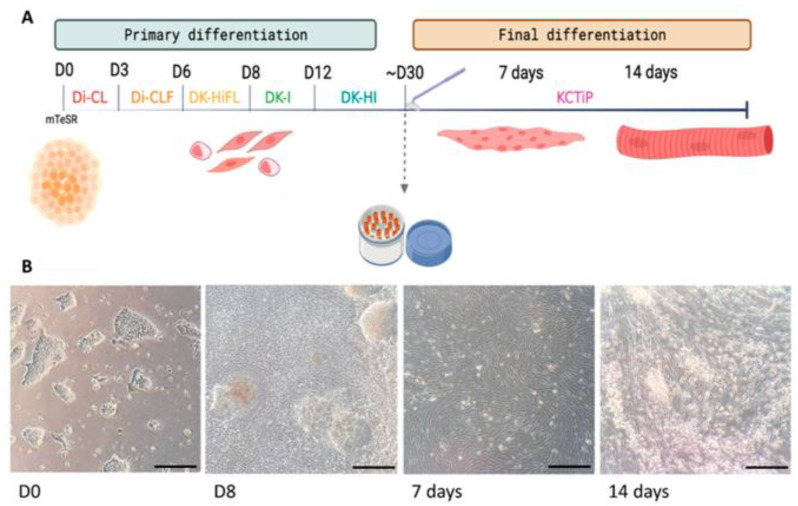
Differentiation of iPSCs towards skeletal muscle. (**A**) Schematic representation of the skeletal muscle differentiation protocol. This process consists of two steps: a primary differentiation, lasting approximately 30 days, and a final differentiation, which takes 7 to 14 days. (**B**) Representative images of cultures taken during the differentiation process at days 0 and 8 of primary differentiation and at 7 and 14 days of final differentiation where the change in morphology of the cells can be noticed. Scale bars: 1 mm.

When the cultures reached 20% confluence, the differentiation process (D0) started. From here onwards, cells were supplemented with consecutive media changes, following the protocol described by Chal et al. [11] for 3 weeks. The sequence of media changes is detailed in Figure 1A.

#### 2.3.2. Expansion and Cryopreservation of Myogenic Progenitors

In order to generate a stock of myogenic progenitors, cultures of 30–32 days are dissociated with Accutase and replated at a 1:5 ratio on new Matrigel-coated dishes with a medium whose composition was developed in our laboratory, VkGM. The composition of this medium is shown in Table 1. At this point, cells can also be cryopreserved in a foetal bovine serum (FBS) with 20% DMSO (Sigma Aldrich, St. Louis, MO, USA, D2650-100ml).

#### 2.3.3. Final Differentiation

At day 32, the primary myogenic cultures were induced to final differentiation following the protocol described by Al Tanoury et al. [12]. To this end, 100.000 cells/cm^2^ were seeded onto Matrigel-coated dishes with the VkGM medium supplemented with 10 μM of the Rock inhibitor. When confluence was near 90%, cultures are induced to differentiate by the addition of the differentiation medium (KCTiP) for 7 or 14 days [12]. The differentiation medium was refreshed every other day.

### 2.4. Characterization of Myogenic Cells

#### 2.4.1. RT-qPCR

The expression of the myogenic markers *MYOD1*, *PAX3*, *PAX7*, *MYH2*, *MYH3* and *MYH8* was quantified by qPCR analysis using the primers listed in Table 2. For this purpose, the total mRNA was extracted at days 8, 16 and 24 of primary differentiation and at days 7 or 14 days of final differentiation. The isolation was performed using the TRI Reagent solution (Invitrogen, Waltham, MA, USA, AM9738), and cDNA synthesis was achieved using the Thermo Scientific RevertAid RT Kit. For the qPCR analysis, an Applied Biosystems^TM^ 7500 Fast Real-Time PCR System was used. All the expression values were normalized to the *GAPDH* gene. Plots are representative of at least three independent experiments.

#### 2.4.2. Immunofluorescence of Myogenic Cells

To detect the presence of typical myogenic markers, an immunofluorescence assay was performed. For this purpose, cells are firstly washed with PBS 1× and fixed with a Formalin solution overnight (Sigma, USA, HT501128-4L). Then, cultures are permeabilized with PBS 1×, 3% Bovine Serum Albumin (BSA) (VWR, Radnor, PA, USA, 0332-100G) and 0.4% Triton-X100 for 1 h. Primary antibody incubation took place overnight and secondary antibody incubation took place for 1 h at room temperature. Finally, cells were counterstained with DAPI (1:1000) and mounted on slides with Prolong (ThermoFisher, USA, P36961). The antibodies and dilutions are listed in Table 3.

### 2.5. Validation of the Human iPSC-Based Model of McArdle Disease

#### 2.5.1. *PYGM* Gene Expression Analyses

For the expression analysis of *PYGM*, a multiplex TaqMan-qPCR analysis was performed using the TaqMan^TM^ gene expression master mix (ThermoFisher, USA, 4369016) and specific *PYGM*-FAM (assay ID #Hs00989942_m1, 10794597) and *GAPDH*-VIC as housekeeping genes for the normalization of the expression levels (assay ID #Hs02786624_g1, 11957021) (Applied Biosystems, Waltham, MA, USA). Plots are representative of at least three independent experiments.

#### 2.5.2. Western Blot

In order to determine the presence or absence of myophosphorylase, a Western blot assay was performed. At days 7 and 14 of the skeletal muscle final differentiation, cultures were scrapped and centrifuged at 300× *g* 5 min. Protein extraction was performed by adding a cell lysis buffer (Tris-HCl mM pH 7,5, NaCl 150 mM, EDTA 5 mM and SDS 0.1%) with protease inhibitors (Roche, Basel, Switzerland, 11873580001). Protein quantification was accomplished using a DC^TM^ protein assay kit, following the instructions of the manufacturer (BioRad, Hercules, CA, USA, 5000112). Subsequently, we performed an SDS-PAGE in 4–20% Mini-PROTEAN^®^ TGX™ precast protein gels (Bio-Rad, USA, #4561094) and transferred the proteins to a nitrocellulose membrane using the Trans-Blot Turbo Transfer system (BioRad, USA, 1704158). After incubation with primary antibodies for 17 h at 4 °C, we incubated the membranes with the GARPO secondary antibody (Molecular Probes, USA, G21234) 1:2500 for 1 h at room temperature. Protein detection was performed with a Clarity Max™ Western ECL Blotting Substrate (BioRad, USA, 1705062). α-tubulin-HRP (Abcam, UK, ab40742) was used as a loading control at 1:5000 dilution.

#### 2.5.3. PAS Staining

The staining method of Periodic acid-Schiff (PAS) is widely used to detect polysaccharides, such as glycogen, in histological and cytological samples. In order to detect glycogen accumulation in the skeletal muscle fibres generated by the differentiation of iPSCs, they were fixed at day 7 of the final differentiation with 3.7% Formaldehyde in 90% ethanol for 1 h at 4 °C. Then, slides were treated with 1% Periodic Acid (Sigma Aldrich, USA, P0430-25G) for 5 min and washed with tap water for 1 min and sterile water for 5 s. Incubation with Schiff’s reagent (Sigma Aldrich, USA, 3952016-500ML) was performed for 15 min at room temperature. After this time, cells are rinsed with sterile water and then with tap water for 10 min in agitation. Finally, cells were mounted with Prolong (ThermoFisher, USA, P36961). Images were obtained with a fluorescence microscope (ZEISS AX10, Jena, Germany). To determinate if the deposits detected by PAS staining were of glycogen origin, a previous permeabilization step was performed, following the protocol published by Schaart et al. [13].

## 3. Results

### 3.1. Generation of a Human iPSC-Based Skeletal Muscle Model of McArdle Disease

In order to assess the pathology in cells more relevant to the disease, we derived myogenic cells from the iPSCs previously generated in our group from a patient with McArdle disease (McA2.7) carrying the homozygous pathogenic variant NM_005609.4: c.2392T>C (p.Trp798Arg) [10] and from a healthy subject (NSV44.1) [9]. For this purpose, a two-step differentiation protocol was followed. For this, iPSC cultures were fed, for 30 days, with sequential media (Di-CL, Di-CLF, DK-HiFL, DK-I and DK-HI), each of them supplemented with a combination of small molecules trying to mimic the embryonic development to paraxial mesoderm [11]. A schematic representation of the differentiation process is illustrated in Figure 1A. First, while maintained in the mTeSR1^TM^ medium (D0), the iPSCs showed bright defined borders (Figure 1B). Once the differentiation process began, the morphology of the colonies started to change, and myogenic precursors emerged beneath the borders of the colonies (Figure 1B; D8). At day 30, the myogenic precursors were expanded in the VkGM medium and cryopreserved, without losing their myogenic capacity. To further generate mature skeletal myofibres, cultures were subjected to a final differentiation for 7 or 14 days. During this time, cultures underwent a characteristic change in the morphology, evidenced by the fusion and enlargement of myoblasts and the alignment of myofibres (days 7 and 14) (Figure 1B).

**Figure 2:**
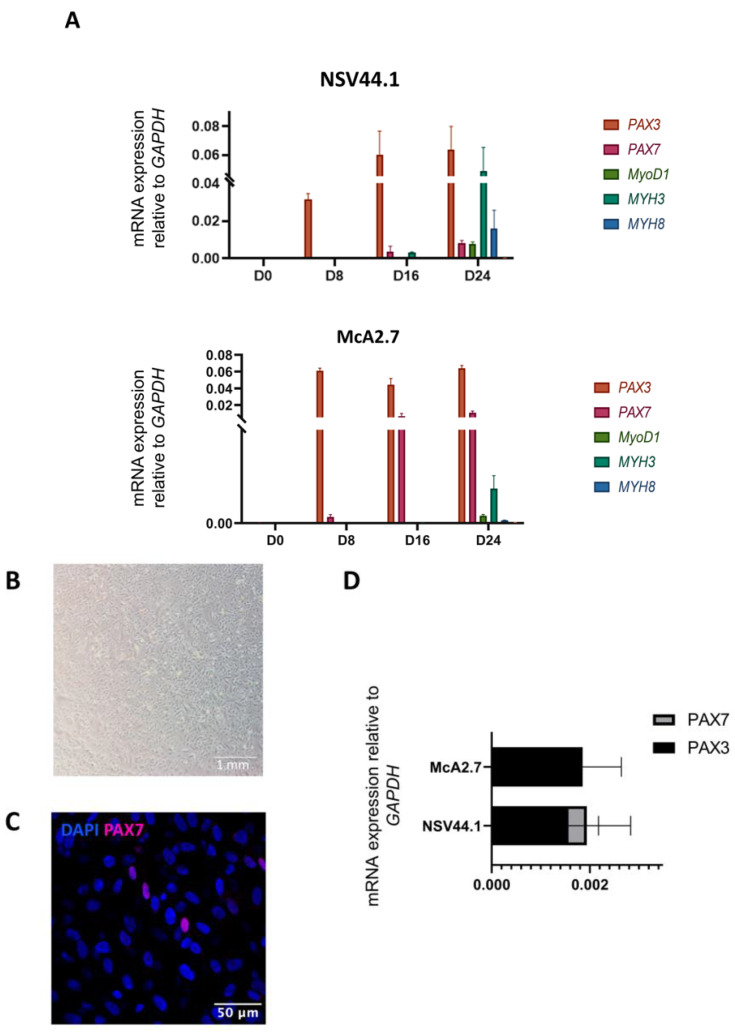

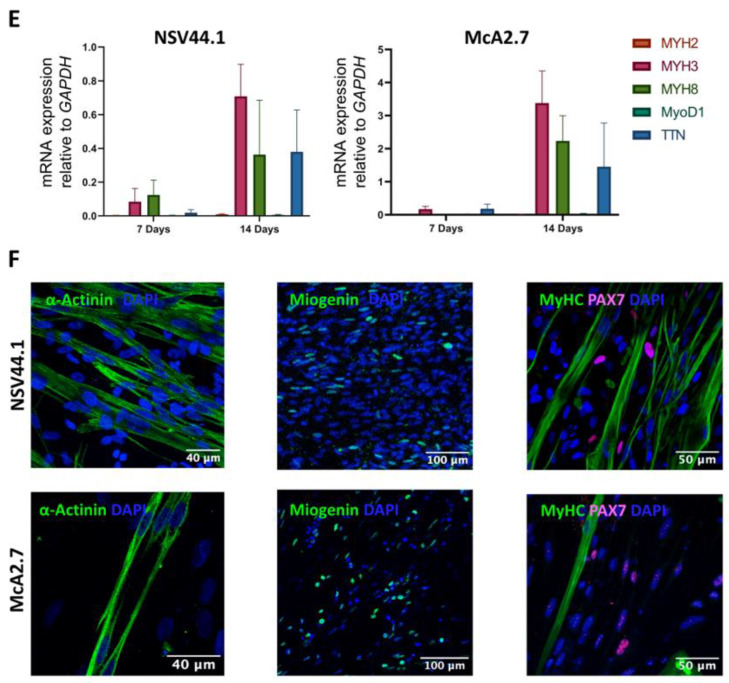
Characterization analyses of the differentiation process from iPSCs to skeletal muscles. (**A**) RT-qPCR analysis used to assess the primary differentiation process. The *PAX3*, *PAX7*, *MYOD1*, *MYH3* and *MYH8* genes were analysed at days 0, 8, 16 and 24 of the primary differentiation process. Values represent the mean of at least three independent replicates and are relative to the expression of the constitutive gene *GAPDH*. Error bars show the standard deviation. (**B**) Brightfield microscope image showing myogenic progenitors. Scale bar: 1 mm. (**C**) Immunofluorescence confocal microscopy analysis of myogenic progenitors. Some cells positive for the *PAX7* marker are visible. Scale bar: 50 μm. (**D**) Diagram of the relative expression of *PAX7* and *PAX3* mRNA in myogenic progenitors. Values represent the mean of at least three independent replicates and are relative to the expression of the constitutive gene *GAPDH*. Error bars show the standard deviation. (**E**) RT-qPCR analysis used to assess the final differentiation process. The *MYH2*, *MYH3*, *MYH8*, *MYOD1* and *TTN* genes were analysed at days 7 and 14 of the final differentiation process. Values represent the mean of at least three independent replicates and are relative to the expression of the constitutive gene *GAPDH*. Error bars show the standard deviation. (**F**) Immunocytochemical analyses of muscle fibres obtained by the differentiation of iPSC lines NSV44.1 and McA2.7. The fibres obtained show positive labelling for the markers α-actinin (**F, left**), miogenin (**F, centre**), MyHC and PAX7 (**F, right**). Scale bars: 40, 100 and 50 μm.

Mature myogenic cells were efficiently derived from both the patient and control iPSCs (Figure 2). During the primary differentiation process, specific markers of myogenic commitment (*PAX3*, *PAX7*, *MYOD1*, *MYH3*, *MYH8* and *MYH2*) were examined by RT-qPCR at days 0, 8, 16 and 24 of the primary differentiation. The results obtained show that the differentiation process took place correctly in both the patient and control (Figure 2A). At day 0, no expression of the myogenic markers was detected. At day 8 of the differentiation process, we first noticed the activation of the *PAX3* expression in both the patient- and control-differentiated iPSC lines. *PAX3* is a distinctive marker of dermomyotomal specification, and its expression was maintained throughout the entire differentiation process. Paraxial mesoderm commitment was assessed at day 16 by the expression of *PAX7*, a marker of pre-myoblast and satellite-like cells, which peaked later at day 24. In addition, at day 24 we detected the expression of the muscle regulatory factor *MYOD1* and the embryonic myosin heavy chain *MYH3* (Figure 2A).

From day 30 onwards, the myogenic progenitors were expanded in the VkGM medium (Figure 2B). At this point, we detected cells positively stained for PAX7 by immunofluorescence analysis (Figure 2C). Furthermore, we confirmed that these progenitors maintained the expression of *PAX3* by RT-qPCR (Figure 2D). The co-expression of these two markers is characteristic of a population of dividing myogenic progenitors and is crucial during skeletal muscle development and regeneration [14].

During final differentiation, RT-qPCR analyses revealed the expression of the typical genes of mature myofibres in the patient and control (Figure 2E). Firstly, the expression of the myogenesis transcription factor *MYOD1* was detected after 7 days of final differentiation. The embryonic isoform of the myosin heavy chain, the *MYH3* expression, was maintained at days 7 and 14 in the two differentiated lines. The manifestation of the perinatal isoform *MYH8* increased from day 7 to 14, as well as the expression of titin (*TTN*), a sarcomeric protein characteristic of mature myofibres. Finally, a slight expression of the fast isoform of the myosin heavy chain *MYH2* was detected after 14 days of final differentiation.

As a final point, we observed by immunofluorescence the expression of α-actinin (Figure 2F, left), myogenin (Figure 2F, centre) and the myosin heavy chain (MyHC) proteins (Figure 2F, right), markers of mature fibres as evidenced by the length and the strong striation pattern of the myotubes and fibres. Moreover, a small number of satellite cells were detected by the PAX7 marker staining (Figure 2F, right).

#### Maturation of the Myofibres

**Figure 3:**
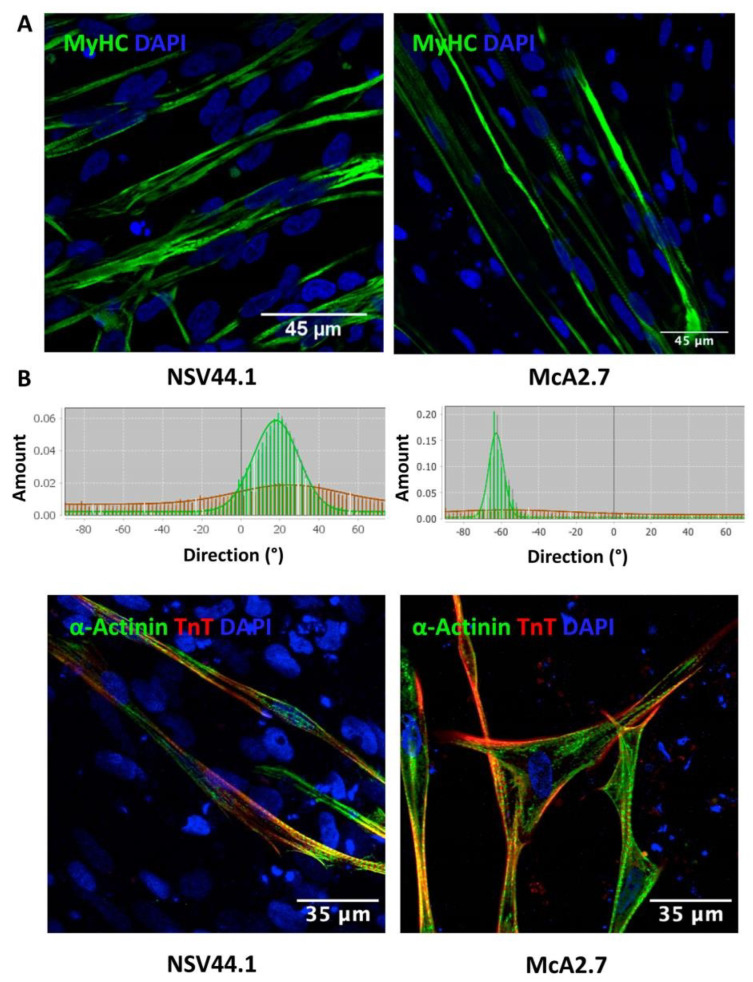
Immunofluorescence analyses to assess typical maturation markers of the myofibres generated by the directed differentiation of the NSV44.1 and McA2.7 iPSC lines. (**A**) Immunofluorescence images of muscle fibres with the MyHC marker highlighting the typical alignment of the obtained myofibres in the differentiation process corroborated with the directionality histograms obtained with the FiJi software (Wayne Rasband NIH, USA). Scale bar: 45 μm. (**B**) Representative immunocytochemical images of the sarcomere structure using α-actinin and troponin-T markers (TnT). Scale bar: 35 μm.

The alignment of the myofibrils in vitro constitutes the main hallmark of the mature skeletal muscle. As it is shown by immunofluorescence with the MyHC marker, the obtained myofibres after the differentiation process were well-aligned. This was also corroborated with the directionality histograms obtained with the FiJi software (Figure 3A) [15].

On the other hand, sarcomere is the functional and structural unit of the mature skeletal muscle. To check the correct organization of the iPSC-derived skeletal muscle sarcomeres, an immunofluorescence analysis was performed using antibodies against the main structural proteins of the sarcomere: α-actinin (Z Disk) and troponin-c. Patterns of sarcomeric localization can be observed in the two differentiated iPSC lines, NSV44.1 and McA2.7 (Figure 3B).

Finally, as another mark of muscle maturity, our healthy iPSC-derived skeletal muscle cultures were able to contract spontaneously (without stimulation) at day 14 of the final differentiation process (Video S1). These contractions were maintained for almost seven weeks. By contrast, no contraction was observed in the skeletal muscle derived from the McA2.7 patient’s iPSCs (*N* = 3) (Video S2).

### 3.2. Validation of the McArdle Disease Skeletal Muscle Model

A set of analyses was performed in order to check if the skeletal muscle model generated from the patient-derived iPSCs mimics the main biochemical features of the disease.

#### 3.2.1. Expression of *PYGM* in iPSC-Derived Skeletal Muscle

**Figure 4:**
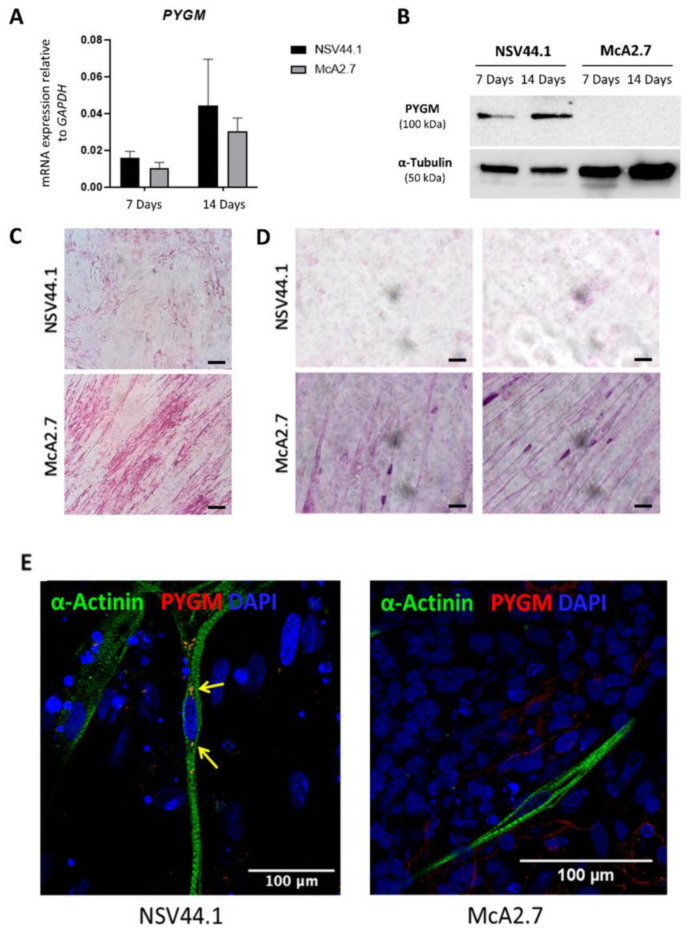
Validation of the McArdle disease skeletal muscle model. (**A**) TaqMan^TM^ assay to assess *PYGM* expression in skeletal muscle fibres differentiated from iPSCs. Both lines show mRNA expression at 7 and 14 days of final differentiation. There are no significant differences according to the Mann–Whitney statistical analysis. Values represent the mean of at least three independent replicates and are relative to the expression of the constitutive gene *GAPDH*. Error bars show the standard deviation. (**B**) Myophosphorylase protein immunodetection assay; α-tubulin was used as the loading control. Analyses demonstrate the presence of PYGM only in the skeletal muscle derived from the NSV44.1 control iPSC line. (**C**,**D**) Representative images of PAS staining of muscle fibres without (**C**) and with (**D**) a previous permeabilization stage (scale bar: 20 μm). The presence of purple clusters around the cell nuclei of McA2.7 fibres is highlighted. A 63× objective. (**E**) Confocal microscopy images showing positive labelling for the myophosphorylase marker PYGM in the NSV44.1 control fibres, indicated by yellow arrows. Scale bar: 100 μm.

Firstly, we analysed and quantified the *PYGM* gene expression by RT-qPCR analysis using Taqman probes, both at 7 and 14 days after the final differentiation. As expected, we detected a higher expression of *PYGM* at 14 days of final differentiation than at 7 days in the two differentiated iPSC lines (NSV44.1 and McA2.7) (Figure 4A). Furthermore, there were no statistically significant differences between the expression of *PYGM* in the skeletal muscle obtained from NSV44.1 and McA2.7, although there is a possible tendency to be slightly more expressed in NSV44.1 versus McA2.7.

#### 3.2.2. Immunodetection Analysis

To further explore the levels of the myophosphorylase protein in the generated skeletal muscle model of McArdle disease, a Western blot analysis after 7 and 14 days of the final differentiation process was performed. In Figure 4B, we can observe the presence of PYGM in the skeletal muscle derived from the control iPSC line NSV44.1, both at 7 and 14 days of final differentiation, but not so in McA2.7 which showed undetectable PYGM levels.

#### 3.2.3. PAS Staining

Another pathognomonic feature of McArdle disease is the abnormal accumulation of glycogen in muscle fibres. To analyse if this occurs in the developed model, a PAS staining was carried out in the differentiated cells. In Figure 4C, it is shown that the McArdle iPSC-derived skeletal muscle cells have higher levels of glycogen than the control. When a permeabilization step was performed prior to the PAS staining, this analysis showed that McArdle muscle fibres tend to accumulate glycogen around the nucleus (Figure 4D). Furthermore, in an immunofluorescence assay, we were able to detect PYGM in the control line NSV44.1 but not in the patient (Figure 4E).

## 4. Discussion

McArdle disease is a rare inherited condition caused by mutations in the *PYGM* gene, which codes for the muscular isoform of glycogen phosphorylase or myophosphorylase [4]. In patients with McArdle disease, the absence of myophosphorylase results in a blockage of energy supply in skeletal muscles, manifesting as exercise intolerance with myalgia, cramps, muscle stiffness and even more severe episodes of rhabdomyolysis and myoglobinuria. To date, there is no cure for McArdle disease and the only treatments available are focused on mitigating symptoms, such as controlled and supervised exercise or pre-exercise nutritional supplementation. Furthermore, although the models available to study the disease have allowed us to better understand its pathophysiological mechanisms and even test possible drugs for its treatment, none of them have been useful for high-throughput pharmacological screening studies [16].

The discovery of induced pluripotent stem cells (iPSCs) has triggered a paradigm shift in personalised medicine due to the possibility of reprogramming any somatic cell back to a pluripotent stage and its subsequent differentiation to any cell type [17]. In addition to being a valuable source of material with the potential for cell therapy applications, iPSCs are a versatile tool for the generation of patient-specific mutation disease models. Recently, in our laboratory, we obtained, for the first time, a human skeletal muscle model of McArdle disease, based on the use of iPSC technology. This model harbours the most frequent pathogenic variant in the Caucasian population in the *PYGM* gene, NM_005609.4: c.148C>T (p.Arg50Ter) [8]. Here, we present the generation of a human iPSC-based skeletal muscle model of McArdle disease carrying the second most frequent pathogenic variant in the Spanish population in the *PYGM* gene, NM_005609.4: c.2392T>C (p.Trp798Arg).

There are two groups of protocols for the differentiation of iPSCs to skeletal muscle [18]: the first involves the overexpression of key transcription factors in myogenic development such as *PAX7* or *MYOG*. These protocols are usually short-lived and have demonstrated a fairly acceptable efficiency of differentiation to myogenic cells. However, the introduction of viral vectors into the cells poses a risk for their future translation to the clinic [19]. The second group of protocols consists of a targeted differentiation by supplementing the culture medium with small molecules that activate or inhibit certain metabolic pathways, thus mimicking embryonic developmental processes [14]. The main advantage of the latter group of protocols is that they are safer for the translation to clinics because they do not require the use of viruses. However, they tend to be long protocols with lower efficiencies and generate heterogeneous cellular populations.

In order to generate a skeletal muscle model of McArdle disease containing the pathogenic variant (p.Trp798Arg), iPSCs from a patient with this disorder and mutation were employed [10]. As a control, we used iPSCs obtained from a healthy person [9]. For the creation of the McArdle muscle model, we implemented a differentiation method that combines two different protocols [11,12], both based on the supplementation of the culture media with small molecules. The first phase of the protocol recapitulates the early stages of paraxial mesoderm differentiation and early myogenesis. During the first eight days of the protocol, a dual modulation of two signalling pathways, key to the generation of large numbers of paraxial mesoderm progenitors and their subsequent specification into the dermomyotome, was induced. This process is characterised by a marked elevation of the *PAX3* expression levels in the cells. By qPCR, we observed that all differentiated lines were found to express *PAX3* from day 8 onwards. Next, factors involved in satellite cell activation and differentiation of presomitic progenitors towards a myogenic fate induction such as the hepatocyte growth factor (HGF) and the insulin-like growth factor (IGF-1) were incorporated. From day 16 of the differentiation, the cells began to express typical markers of early myogenesis such as *PAX7*, *MYOD1* and *MYH3*, which reached peak expression at day 24.

From day 30 onwards, the expansion and cryopreservation of myogenic progenitors could be performed. This was key to the generation of the skeletal muscle model because of the availability of a stock of frozen cells for later applications. Moreover, we developed a culture medium for the progenitors’ expansion (VkGM) based on the main components of the commercial media and the work published by Jarocha et al. [20]. The myogenic progenitors in VkGM were positively labelled for PAX7 by the immunofluorescence and co-expression of the *PAX3* and *PAX7* genes. This fact not only demonstrates that cells maintained their myogenic character after thawing but also that they were able to resist passaging, grew properly and that cultures were enriched in myogenic progenitors.

The second stage of the protocol consisted of the final differentiation to adult skeletal muscle fibres. To induce the myoblast differentiation to mature muscle fibres, the culture medium was supplemented with CHIR99021, an agonist of the Wnt pathway and knock-out serum. In addition, SB-431542, an inhibitor of the transforming growth factor type β (TGF-β) that enhances myofibre fusion efficiency [21], and prednisolone, a synthetic glucocorticoid known to promote myogenic differentiation [22], were added. The muscle fibres obtained from both the patient and control showed elevated expression values of *MYH3*, *MYH8* and *TTN*, all of which are typical markers of muscle fibres. They also stained positively for the muscle fibre markers α-actinin, myogenin, myosin heavy chain (MyHC) and the satellite cell marker PAX7.

One of the main problems to be addressed when differentiating to myogenic cells from iPSCs is the lack of maturation that is achieved, in most cases, with in vitro differentiation protocols. The fact that the myotubes obtained do not reach a sufficiently mature phenotype makes it difficult to generate appropriate disease models for those pathologies that manifest themselves at advanced stages of development [23]. Remarkably, the differentiation method used in this work resulted in aligned multinucleated fibres with a defined sarcomeric structure that also showed spontaneous contractions (without the need of an external stimulation). This could be explained due to the presence of motor neurons in the cultures. In vivo, for a contraction to occur, there must first be a stimulation of the skeletal muscle in the form of an action potential from a motor neuron. Myofibers and motor neurons in close proximity connect with each other to form the functional neuromuscular junctions [24]. Yet, it is highly challenging to exclusively differentiate iPSCs into a single desired cell type. In consequence, most of the differentiation protocols generate a heterogeneous cellular population [25]. This could explain the presence of motor neurons in our cultures and the generation of an innervated mature skeletal muscle model that is able to contract. Further studies are needed to address whether the observed contractions are really associated with nervous stimuli.

As a final point, we confirmed that the generated patient iPSC-based skeletal muscle model mimicked the main biochemical aspects of McArdle disease. To this aim, we firstly verified that both skeletal muscles obtained from the control and patient iPSCs showed *PYGM* expression. These results are consistent with previously published data where patients with missense variants presented mRNA levels similar to or at least up to 40% of the healthy controls [26]. Another distinguished hallmark of patients with McArdle disease is the absence of the myophosphorylase protein in muscles. Immunoblots from the patients harbouring premature termination codon-predicting mutations show a total absence of the myophosphorylase protein. Remarkably, this finding is also true for the majority of patients harbouring missense mutations in *PYGM* (including the mutation of c.2392T>C (p.Trp798Arg)), in whom the normal protein translation and biosynthesis of PYGM would be theoretically expected [26]. It thus seems likely that the suppression of the myophosphorylase expression is a commonly elicited mechanism in the muscle tissue of these patients. To check if the generated model also fulfilled this criterion, the presence of PYGM was analysed by both immunodetection and immunofluorescence, confirming the lack of myophosphorylase in the McArdle iPSC-derived skeletal muscle model and its presence in the control one. We have also corroborated that the patient iPSC-based model showed a glycogen accumulation by PAS staining. An abnormal glycogen accumulation in muscle fibres is another pathognomonic feature of McArdle disease and is produced due to the inability of these patients to break down glycogen as a consequence of the myophosphorylase deficiency [2]. In fact, PAS staining is routinely performed in muscle biopsies from McArdle patients for the clinical diagnosis of the disease [27]. Finally, it should be noted that the spontaneous contraction, without the need of a stimulation, of the differentiated skeletal muscle was only detected in the control but not in the patient. This is consistent with previously reported observations that showed a reduced muscle contraction force in McArdle patients as a consequence of a reduced energy-producing capacity [28].

In summary, we report here the first human iPSC-based skeletal muscle model of McArdle disease carrying the second most frequent pathogenic variant in the *PYGM* gene in the Spanish population: NM_005609.4: c.2392T>C (p.Trp798Arg). As far as we know, this is the only human cellular model of McArdle disease for a missense variant in *PYGM* generated with iPSC technology. The created model mimics the main biochemical hallmarks of this disorder, and its use as a platform for the search of a personalized therapy against this disorder will be very helpful.

## Figures and Tables

**Table 1:** Composition of the VkGM medium.

	Concentration	Company and Reference
DMEM/F12		ThermoFisher, USA, 11320-082
FBS	18%	Sigma Aldrich, USA, F7524-500ML
Insulin	1%	Sigma Aldrich, USA, I9278
P/S	0.2%	Sigma Aldrich, USA, P0781
Human recombinant EGF	20 ng/mL	Stemcell technologies, Canada, 78006.1
Human bFGF-2	20 ng/mL	Miltenyi, Germany, 130-093-839
Human recombinant HGF	10 ng/mL	Stemcell technologies, Canada, 78019.1
Dexamethasone	10 µM	Sigma Aldrich, USA, D4902

**Table 2:** Primers used in the qPCR analysis for myogenic cell characterization.

Target	Forward/Reverse Primer (5′-3′)
*MYOD1*	GACGGCATGATGGACTACAG/AGGCAGTCTAGGCTCGACAC
*PAX3*	TACAGGTCTGGTTTAGCAAC/GATCTGACACAGCTTGTGGA
*PAX7*	CAGACAGGTGGCGACTCC/CGCGGCTAATCGAACTCAC
*MYH2*	GGAGCTGGTGGAGGGGCCAA/TGCTCCATGGCACCAGGAGTTT
*MYH3*	GCTTGTGGGCGGAGGTCTGG/AGGGCTGGTTCTGAGCCTCGAT
*MYH8*	TCCACCAAGAACCCAGAGAGTGG/TGGGCCTCAATCCGCTCCTT
*TITIN*	CCGAAATGCATCAGTCAGCG/CCTTGCAAGCTTGTGTCACC

**Table 3:** Antibodies used for immunofluorescence analyses of myogenic cells.

Antibody	Dilution	Company and RRID
Rabbit anti PAX7	1:50	Abcam, Cambridge, UK, (ab187339) RRID: AB_2813893
Mouse anti Myogenin	1:25	Abcam, UK (ab212668) RRID: AB_262133
Mouse anti α-Actinin	1:500	Sigma Aldrich, USA, (A7811) RRID: AB_476766
Mouse anti MyHC	1:250	Sigma Aldrich, USA, (M4276) RRID: AB_477190
Rabbit anti PYGM	1:50	Sigma Aldrich, USA, (HPA056003) RRID: AB_2683006
Goat anti Mouse IgG (H + L), Alexa Fluor 488	1:300	ThermoFisher, USA, (A-11029) RRID: AB_2534088
Goat anti Rabbit IgG, Alexa Fluor 568	1:1000	Invitrogen, USA, (A-11011) RRID: AB_143157

## Data Availability

The data presented in this study are available on request from the corresponding author.

## Supplementary Materials

The following supporting information can be downloaded at: https://www.mdpi.com/article/10.3390/biomedicines11092434/s1, Video S1: Contraction capacity of the iPSC line NSV44.1 at day 14 of the final differentiation process; Video S2: Contraction capacity of the iPSC line McA2.7 at day 14 of the final differentiation process.
